# Supplementary material for: Resistance to Aflatoxin Accumulation in Maize Mediated by Host-Induced Silencing of the Aspergillus flavus Alkaline Protease (alk) Gene
Source: J Fungi (Basel). 2021 Oct 26;7(11):904. doi: 10.3390/jof7110904 (PMC8622731; doi:10.3390/jof7110904)
Supplement: Supplementary file 1 [file jof-07-00904-s001.zip › jof-1415096-supplementary.pdf]

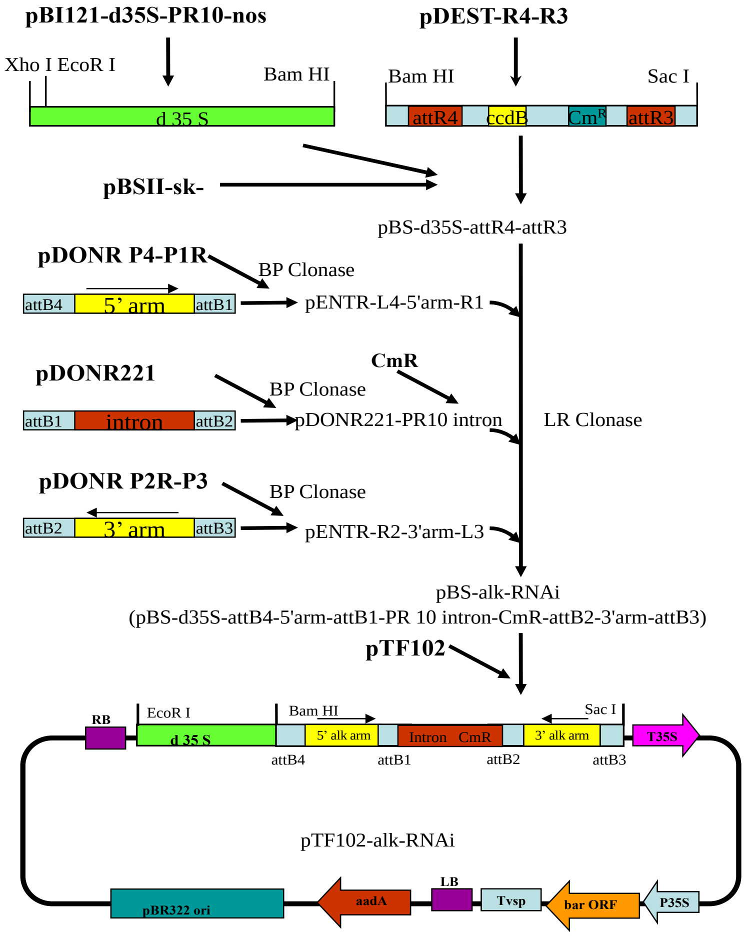

Figure S1. Construction scheme of gene silencing vector. The pBS- d35S- R4-R3 vector containing a double 35S promoter, followed by an attR4-ccdB-CmR-attR3 cassette amplified from pDEST^{TM} R4-R3 (Invitrogen, Carlsbad, CA) in the pBluescript SK- was constructed in an earlier study Chen et al., [37]. The DNA regions corresponding to the *alk* 5 ′arm and 3′arm were amplified by PCR with primers containing unique homologous recombination sites cloned into their corresponding entry vectors. They were recombined with pDONR221-PR 10-intron-CmR containing a chloramphenicol resistance gene (CmR) selection marker in the middle of the PR10 intron through the LR clonase reaction to assemble the RNAi cassette into the pBluescript vector to produce the pBS-d35S-attB4-5′arm- attB1-PR10 intron-CmR-attB2-3′arm-attB3 vector (named pBS-II-Alk-RNAi). The RNAi cassette was then cloned into the pTF102 vector through ligation to produce the final pTF102- alk-RNAi vector. This figure was modified from Chen et al. [37].

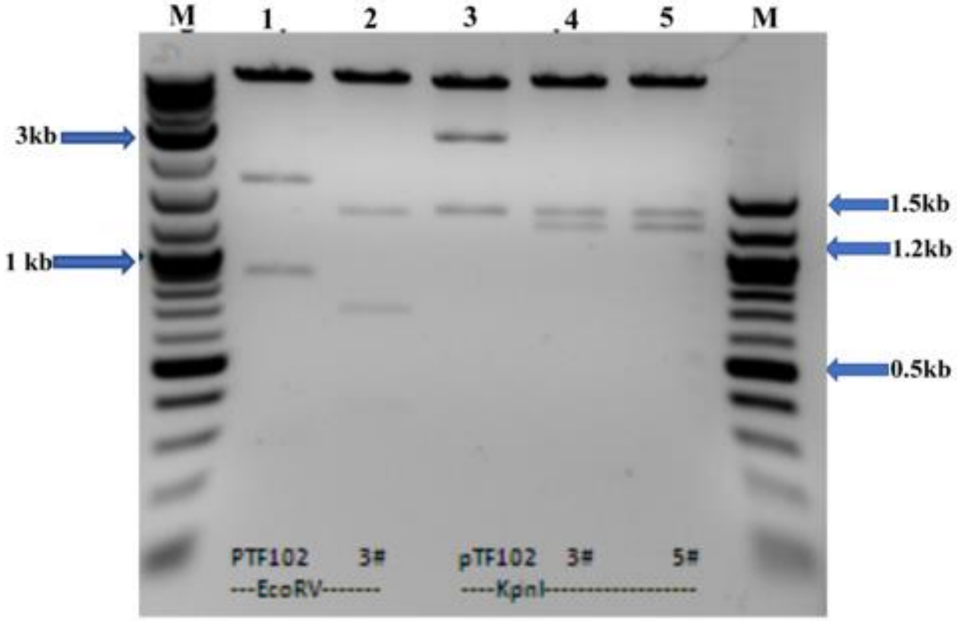

Figure S2. Restriction digestion of the pTF102-Alk-RNAi construct to confirm its correct assembly. Lanes 1 and 3 are the original pTF102 vector digested with EcoR V and Kpn I, which resulted in the expected sizes of 8.9, 1.7, 0.88, 0.23 kb and 7.7, 2.5 and 1.3 kb, respectively; lanes 2, 4 and 5 are two independent clones of pTF102-Alk-RNAi vector digested with EcoR V and Kpn I, which resulted in the expected sizes of 8.7, 1.3, 1.1, 0.29 kb; and 1.28, 1.35kb, respectively.

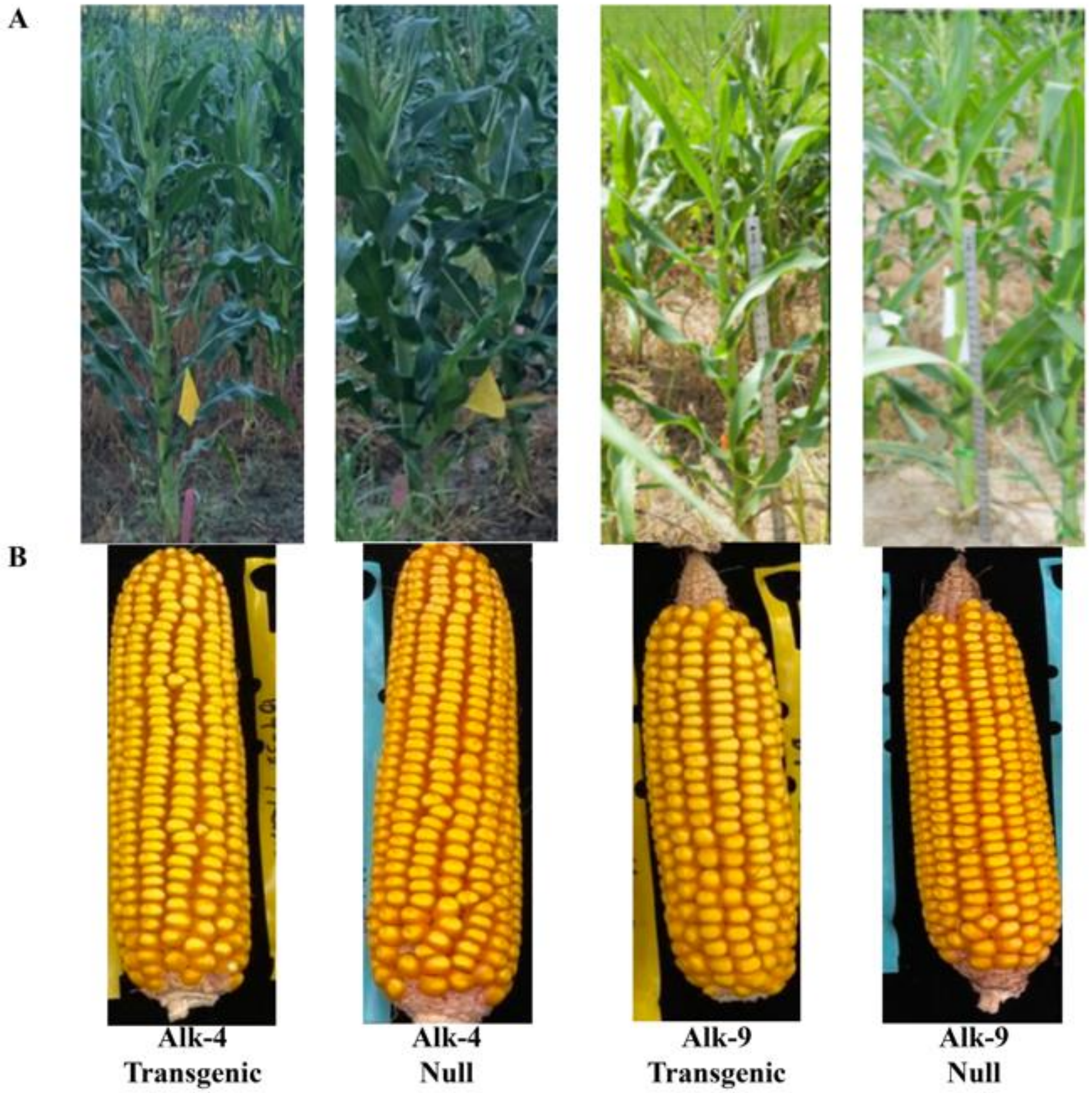

Figure S3. Representative appearance of plant morphology (A) and kernel set (B) of Alk-RNAi transgenic and null of T4 generation plants at flowering and ears at harvest.

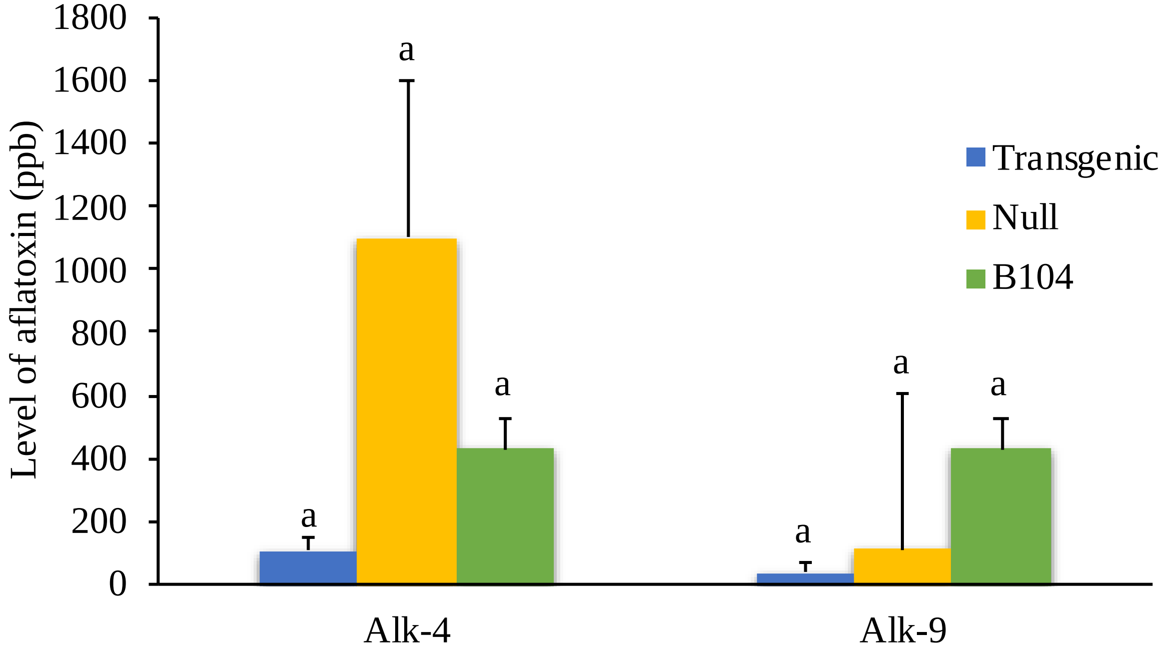

Figure S4: Aflatoxin production in the T4 generation transgenic and null kernels of Alk-4 and Alk-9 events and B104 in 2017 under field inoculation conditions. Data shown are the means from each event (3-4 replicates per ear, and 5 to 10 ears per event). Bars with same letters are not significantly different at $P \leq 0.05$. 4×10^5 conidia/mL inoculum concentration was used for this field inoculation study. Four different points were inoculated on each ear.

Table S1. List of primers and probes used for genotyping and zygosity test of the Alk-RNAi events

Primer Name	Oligonucleotide sequence (5′→3′)
Alk-F	GCG TTA CCG TTG TAG GCA AG
Alk-R	TCC AGA AGA GCA ACA ACC GC
RT-Alk-F	ATC CGC TCG CAT TTT TGA AT
RT-Alk-R	AGA TCT GGT CCT CCT CGA CGT A
RT-Alk-F (Taq)	CTC GCT GCC CTT GAG AAC
RT-Alk-R (Taq)	GCT GCC CTT AAC ATC CTT GA
aattB4-Alk-F	*GGGACAACTTTGTATAGAAAAGTT*TCACTGTTGCCGCTATCC
aattB1-Alk-R	*GGGACAACTTTGTATAGAAAAGTT*TGTAGGCAAGCAGGTTAGGG
aattB2-Alk-F	*GGGACAACTTTGTATAGAAAAGTT*GCGTTACCGTTGTAGGCAAG
aattB3-Alk-F	*GGGACAACTTTGTATAGAAAAGTT*TCCAGAAGAGCAACAACCGC
Alk-probe*	FAM/CAA CTC CTT/ZEN/GAT GCG CTT GGTCAC/3IABkFQ
RT-Adh1-F(Taq)	CGT CGT TTC CCA TCT CTT CCT CC
RT-Adh1-R(Taq)	CCA CTC CGA GAC CCT CAG TC
Adh-probe*	HEX-AATCAGGGC/ZEN/TCATTTTCTCGCTCCTCA-IBFQ
RT-Zm18S-F	GAGAAACGGCTACCACATCCA
RT-Zm18S-R	ACGCGCCCGGTATTGTTAT
Bar-F	TGCACCATCGTCAACCACTACATCGAG
Bar-R	CAGGTGAAGTCCAGCTGCCAGAAAC
RT-Bar-F (Taq)	GGA AGT TGA CCG TGC TTG T
RT-Bar-R (Taq)	GAT CTA CCA TGA GCC CAG AAC
Bar-probe*	FAM-CGATGTAGT/ZEN/GGTTGACGATGGTGCA-IBFQ
RT-ZmMEP-F	TGTACTCGGCAATGCTCTTG
RT-ZmMEP-R	TTTGATGCTCCAGGCTTACC
β-Tubulin- F	TCTTCATGGTTGGCTTCGCT
β-Tubulin- R	CTTGGGTCGAACATCTGCT

Italics indicate the homologous recombination sites that is added to the end of the gene specific primer sequences. *: the probes were labeled with FAM (6-fluorescein) or HEX (hexachloro fluorescein) at the 5′end and double quenched with ZEN and Iowa Black FQ (IBFQ) or ZEN and IBFQ.

Table S2. List of transformation events received from the Iowa State University transformation facility.

Line name	Total number of kernels	Average kernel weight (g)
Alk-1	143	0.1972
Alk-2	126	0.2333
Alk-3	24	0.2706
Alk-4	54	0.2369
Alk-5	116	0.2362
Alk-6	52	0.2364
Alk-7	108	0.2138
Alk-8	49	0.2774
Alk-9	258	0.1775
Alk-10	116	0.2646
Alk-11	113	0.2274
Alk-12	309	0.1799

Table S3. Aflatoxin production in the kernels of selected T1 Alk events using the kernel screening assay

Event		Number of kernels	Mean aflatoxin level (ppb)	P-value
	Null	6	593,891	
Alk-3	Transgenic	4	412,103	0.19
	Null	9	286.147	
Alk-4	Transgenic	4	71,347	0.03
	Null	7	465,318	
Alk-9	Transgenic	4	25,2815	0.05
